# Vosoritide (Voxzogo) for Achondroplasia: A Review of Clinical and Real-World Evidence

**DOI:** 10.7759/cureus.87983

**Published:** 2025-07-15

**Authors:** Hansan L Jones, Theodore I Nania, Julian M Moore, Jeffrey D Reed, Alec W Lyons, Pamela Potter, John Ashurst, Heather Holley

**Affiliations:** 1 Research, Midwestern University Arizona College of Osteopathic Medicine, Glendale, USA; 2 Pharmacology, Midwestern University Arizona College of Osteopathic Medicine, Glendale, USA; 3 Pediatrics, Midwestern University Arizona College of Osteopathic Medicine, Glendale, USA

**Keywords:** achondroplasia, fgfr3, growth hormone alternatives, rare autosomal dominant skeletal dysplasia, skeletal dysplasia, vosoritide

## Abstract

Achondroplasia is the most common genetic skeletal dysplasia, caused by activating mutations in the FGFR3 gene that impair endochondral ossification and result in disproportionate short stature. Vosoritide (VOXZOGO®), a C-type natriuretic peptide analog, is the first targeted therapy approved for achondroplasia and acts by antagonizing FGFR3 signaling to promote bone growth. This review evaluates current clinical evidence on the efficacy, safety, and long-term potential of vosoritide in children with achondroplasia. A structured literature search of PubMed and Embase was conducted using the terms “Achondroplasia” and “Vosoritide” or “Voxzogo”. We included clinical trials and extensions, observational studies, real-world reports, and case reports published between May 2018 and May 2025 while excluding articles that focused on guidelines for the clinical application of Vosoritide. Phase II and III trials indicate that daily subcutaneous vosoritide (15 μg/kg) increases annualized growth velocity (AGV) by approximately 1.5-2.0 cm/year compared to placebo. Extension studies have demonstrated sustained growth over seven years. Twelve-month retrospective studies from France, Germany, and Japan have independently observed similar increases in linear growth. Trials also report improvements in body proportions and craniofacial development. At the same time, retrospective studies have shown a reduction in lumbar lordosis and leg bowing, as well as improvement in six-minute walk distance and potential benefit in daily function. Common adverse effects include mild injection site reactions and transient hypotension in infants. Treatment with vosoritide may improve long-term outcomes for children with achondroplasia when compared with the current pharmacological standard of care. Ongoing studies are expected to clarify its effects on adult height, potential effects on skeletal deformities, and overall quality of life.

## Introduction and background

Achondroplasia is the most common form of disproportionate short stature. It is characterized by rhizomelic limb shortening, macrocephaly, and midface hypoplasia, which can lead to significant medical complications, including foramen magnum and spinal stenoses, sleep apnea, and recurrent otitis media [1]. In addition to these physical challenges, individuals with achondroplasia often experience functional limitations and psychosocial difficulties, which impact their overall quality of life [2].

Achondroplasia is caused by a gain-of-function mutation in the fibroblast growth factor receptor 3 (FGFR3) gene, which encodes a transmembrane tyrosine kinase receptor that plays a role in chondrocyte proliferation and differentiation. Wild-type FGFR3 responds to fibroblast growth factor (FGF) in a ligand-dependent manner to activate the RAS-MAPK pathway. Mutations in FGFR3, including those observed in achondroplasia, result in a cell surface receptor that is both ligand-dependent and ligand-independent. The ultimate consequence of this signaling cascade is the excessive inhibition of intracellular messages that stimulate chondrocyte proliferation and bone matrix synthesis, leading to decreased length of the long bones and abnormalities in other bones [3-6]. This dysregulated signaling leads to the characteristic features of achondroplasia [7].

Historical treatment strategies addressed the symptoms but not the underlying molecular defect associated with achondroplasia. Growth hormone (GH) therapy is approved in several countries for the treatment of achondroplasia and has demonstrated some efficacy in improving vertical growth during the first year of treatment. However, the efficacy of GH therapy remains controversial and is not universally accepted as a consensus outcome [8]. GH therapy has several drawbacks, including the risk of spinal cord compression, worsening of anteroposterior spinal curvature defects, risk for acromegaly, and the burden of daily injections [8,9]. Surgical limb-lengthening procedures have been used as an alternative to pharmaceutical therapy but involve a long recovery period and substantial risks, including unplanned reoperation, nerve damage, fracture after removal of hardware, and infection [10-12].

Vosoritide (VOXZOGO®) is the first pharmacological therapy that targets the underlying molecular mechanism of achondroplasia [7]. By binding to natriuretic peptide receptor B (NPR-B) on the surface of chondrocytes, vosoritide stimulates the production of intracellular cyclic guanosine monophosphate (cGMP), leading to the inhibition of aberrant FGFR3 signaling [13]. Thus, vosoritide restores balance to the intracellular messengers that drive endochondral ossification [13], a process critical to longitudinal bone growth, by allowing chondrocytes in the growth plate to proliferate and differentiate appropriately. Following the announcement of the first Phase III trial results in December 2019, clinical trials have continued to demonstrate significant improvements in annualized growth velocity (AGV) and skeletal proportionality possible via vosoritide-mediated FGFR-3 modulation, leading to FDA approval of the drug for public use in November 2021 [7,14,15]. This review aims to present the current evidence on vosoritide, focusing on its efficacy, safety profile, and real-world clinical application [7].

## Review

Methods: literature search and selection criteria

**Figure 1:**
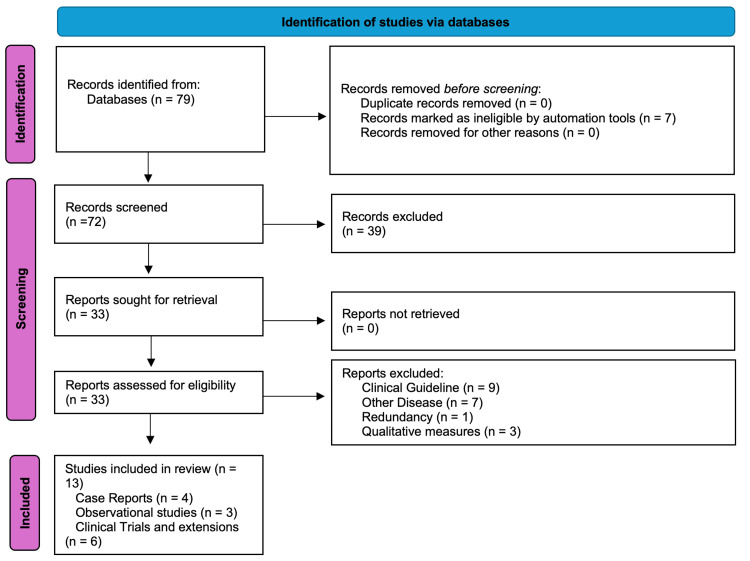
PRISMA flow diagram of study selection PRISMA: Preferred Reporting Items for Systematic Reviews and Meta-Analyses

A PubMed (MEDLINE) and Embase literature search was conducted from May 1, 2018, through May 31, 2025, using keywords and medical subject headings related to achondroplasia and vosoritide. The primary search terms included “achondroplasia” and “vosoritide” or “Voxzogo”. A secondary search was performed using the first and last author names of major studies in the field. Clinical trials (Phase II or III) of vosoritide in achondroplasia, open-label extension studies, follow-up reports, observational studies, and case series reporting real-world use of vosoritide were included in the review. Conference abstracts were considered if they provided data not yet published in full manuscripts, though the focus remained on peer-reviewed publications. Trials of other investigational therapies for achondroplasia (e.g., FGFR3 inhibitors), preclinical studies, and publications not presenting original research were excluded from the review. Two authors independently screened the titles and abstracts of all search results. Of the 79 reports identified after the keyword search, seven were excluded because they were published before May 2018, and 39 were excluded based on title and abstract screening. Thirty-three full-text reports were assessed for eligibility; of these, nine were excluded because the text sought to establish clinical guidelines for the use of vosoritide rather than report clinical outcomes. Seven additional studies were excluded due to a focus on types of skeletal dysplasias other than achondroplasia, and three were excluded for reporting only qualitative data (e.g., surveys on caregivers’ perspectives about administering daily vosoritide injections). Thirteen papers were selected for inclusion in the review, consisting of four case reports, three real-world retrospective studies, and six papers describing clinical trials and their extensions (Figure 1).

**Figure 2:**
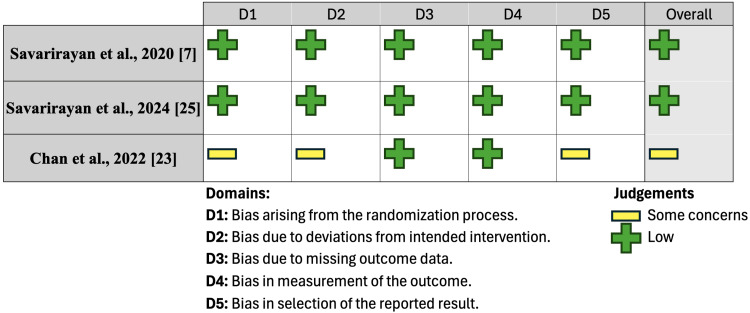
Quality assessment of RCTs by RoB 2.0 RoB 2.0: Cochrane Risk of Bias (RoB) 2.0 tool

**Figure 3:**
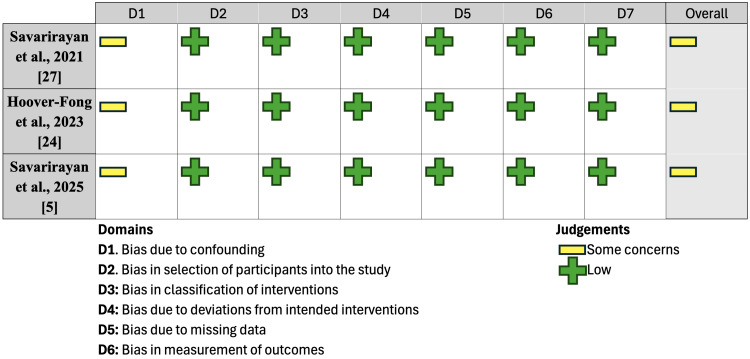
ROBINS-I risk of bias assessment for extension studies ROBINS-I: Risk Of Bias In Non-randomized Studies - of Interventions

To assess the methodological quality of the included studies, randomized controlled trials (RCTs) were evaluated using the Cochrane Risk of Bias (RoB) 2.0 tool; the findings from this analysis can be found in Figure 2. The ROBINS-I risk of bias assessment was used for extensions of these clinical trials, and findings are reported in Figure 3. Observational studies were evaluated using the Newcastle-Ottawa Scale (NOS) and are summarized in Table 1. Case series were assessed using the Joanna Briggs Institute (JBI) critical appraisal checklist for case series, summarized in Table 2, while case reports were evaluated using the JBI critical appraisal checklist for case reports, summarized in Table 3. These assessments were independently conducted by two reviewers. In the event of disagreement, discrepancies were resolved by mutual agreement or consultation with a third reviewer. 

**Table 1:** Quality assessment of observational studies by NOS NOS: Newcastle-Ottawa Scale

Study	Representativeness of the exposed cohort	Selection of the non-exposed cohort	Ascertainment of exposure	Demonstration that the outcome of interest was not present at the start of the study	Compare the ability of cohorts based on the design or analysis	Assessment of outcome (1)	Was the follow-up long enough for outcomes to occur?	Adequacy of follow-up of cohorts
Cormier-Daire et al., 2025 [16]	1	1	1	1	2	1	1	1
Reincke et al., 2025 [17]	1	1	1	1	2	1	1	1
Sawamura et al., 2025 [18]	1	0	1	1	1	1	1	1

**Table 2:** JBI critical appraisal checklist for case series JBI: Joanna Briggs Institute Source: Refs. [19-21] JBI grants the use of these tools for research purposes only.

Checklist Item	Clinton et al., 2025 [19]	Nishioka et al., 2024 [20]
1. Were there clear criteria for inclusion in the case series?	Yes	Yes
2. Was the condition measured in a standard, reliable way for all participants?	Yes	Yes
3. Were valid methods used to identify the condition for all participants?	Yes	Yes
4. Did the case series include consecutive participants?	Unclear	Unclear
5. Did the case series include all eligible participants?	Yes	Unclear
6. Was there clear reporting of the demographics of the participants in the study?	Yes	Yes
7. Was there clear reporting of clinical information of the participants?	Yes	Yes
8. Were outcomes or follow-up results clearly reported?	Yes	Yes
9. Was there clear reporting of the presenting site(s)/clinic(s) demographic information?	Yes	Yes
10. Was statistical analysis appropriate?	N/A	N/A
Overall Appraisal	Include	Include

**Table 3:** JBI critical appraisal checklist for case reports JBI: Joanna Briggs Institute Source: Refs. [22-24] JBI grants the use of these tools for research purposes only.

Checklist item	Ireland et al., 2025 [22]	Gunji et al., 2025 [23]
Were the patient’s demographic characteristics clearly described?	Yes	Yes
Was the patient’s history clearly described and presented as a timeline?	Yes	Yes
Was the clinical condition on presentation clearly described?	Yes	Yes
Were diagnostic tests and results clearly described?	Yes	Yes
Was the intervention(s) or treatment procedure(s) clearly described?	Yes	Yes
Was the post-intervention clinical condition clearly described?	Yes	Yes
Were adverse events (harms) or unanticipated events described?	No	Yes
Does the case report provide takeaway lessons?	Yes	Yes
Overall Appraisal	Include	Include

We organized the results from the included papers into sections covering clinical trial evidence and real-world outcomes. We now present a summary supplemented by tables that concisely compile findings for ease of reference. This analysis provides an up-to-date overview of current knowledge regarding vosoritide therapy in achondroplasia.

Clinical trials of vosoritide in achondroplasia

Phase II and III trials have been conducted to evaluate the efficacy and safety of vosoritide in children with achondroplasia. These trials consistently showed that daily subcutaneous administration of vosoritide can significantly increase AGV while maintaining a favorable safety profile [7]. Table 4 summarizes the findings discussed in this section.

**Table 4:** Clinical trials of vosoritide in achondroplasia mo = months; AE = adverse event; SAE = serious adverse event; AGV = annualized growth velocity; SD(S) = standard deviation (score); SIDS = sudden infant death syndrome

Study	Population	Design (Phase)	Treatment Duration	Key Efficacy Outcomes	Key Safety Findings
Savarirayan et al., 2020 [7]	Children aged 5-1 7 years (n=121)	Randomized, placebo-controlled (Phase III)	One year	Δ AGV: +1.57 cm/year vs placebo (p<0.0001); height z-score ↑ ~0.3 in treatment vs no change in placebo; no change in body proportion at one year.	Mild, transient hypotension in rare cases; injection-site reactions common. No treatment-related serious AEs; no discontinuations.
Chan et al., 2022 [25]	Children aged 5-14 years (n=35)	Open-label, dose-escalation study (Phase II)	Two years	The exposure–response relationships for changes in both annualized growth velocity, saturated at 15 μg/kg.	No evidence of accumulation with once-daily dosing.
Hoover-Fong et al., 2023 [26]	Children aged 5-14 years (n=30)	Open-label extension of dose-escalation study (Phase II)	Mean of 76 months (max 97 months)	Height z-score +1.56 (0.63) at Month 72 and +1.46 (0.69) at Month 84 relative to an untreated ACH population. Significant ↓ in the upper body to lower body ratio.	No new safety issues; no drug-related SAEs. Similar AE profile as initial two years.
Savarirayan et al., 2024 [27]	Infants and children aged 0-4 years (n=75)	Randomized, placebo-controlled– three cohorts 0-<6 mos, 6-23 mos, 24-59 mos. (Phase II)	One year	Height z-score increased +0.25 SD vs placebo (95%CI –0.02 to +0.53) – trend favoring vosoritide, but not statistically significant at one year. Growth velocity nominally higher with treatment; longer follow-up ongoing.	Generally mild AEs. One infant death (SIDS, deemed unrelated). No treatment-related SAEs; AE types similar to older children, including injection-site erythema and mild infections.
Savarirayan et al., 2025 [5]	Children aged 5-17 years (n=119)	Open-label extension (Phase III Extension)	Up to three to five years, with an interim analysis at three years	Treated children were an average of 5.75 cm taller than untreated peers after three years; AGV maintained ~5-6 cm/year. Significant ↓ in the upper body to lower body ratio.	No new safety issues; no drug-related SAEs. Similar AE profile as the initial year.

Phase II Dose-Escalation and Extension Studies

The first Phase II study performed on vosoritide (BMN 111-202) was a dose-escalation, open-label trial conducted in 35 children aged 5-14 years to assess the safety, tolerability, and optimal dosing of vosoritide [25]. Patients received daily subcutaneous vosoritide injections at doses of 2.5, 7.5, 15, and 30 μg/kg for 24 months. Results demonstrated a dose-dependent increase in AGV, with the 15 μg/kg dose saturating the effect on growth velocity [25]. The long-term extension study (BMN 111-205) followed 30 of the original participants for up to seven years, confirming that sustained vosoritide use maintains growth velocity improvements over time with a favorable side-effect profile [26]. These children will continue to be followed until they reach their final adult height.

Phase III Randomized, Placebo-Controlled Trial in 5-17-Year-Olds With Achondroplasia

Following the success of Phase II trials, Savarirayan et al. conducted a randomized, placebo-controlled, double-blind trial evaluating 52 weeks of vosoritide treatment in 121 children with achondroplasia aged 5-17 years [3]. Participants were randomized to receive daily vosoritide 15 μg/kg (n = 60) or placebo (n = 61). The primary endpoint was the difference in AGV after one year of treatment. Children treated with vosoritide showed a statistically significant increase in growth velocity of 1.57 cm/year (8.26 cm/year vs. 6.69 cm/year) [3]. Secondary endpoints in the Phase III trial included height z-score and body proportions. Vosoritide-treated children experienced an increase in height z-score (mean: +0.28 vs. -0.01 in the placebo group) over one year, approaching the growth range of age-matched average-stature children.

Phase III Open-Label Extension Study

All 121 children from the Phase III trial were invited to enter an open-label extension, in which both groups received vosoritide to assess the long-term outcomes of therapy. Results from the trial indicate that growth velocity gains were maintained with continued therapy. After two to three years of therapy, children treated with vosoritide accumulated an average of 5.7 cm of additional height compared to untreated peers in natural history comparisons [5]. A December 2024 update reported a median height gain of ~11 cm after seven years of continued therapy [5]. Notably, the annual growth velocity of treated children during these years approached that of age-matched children without achondroplasia, especially before the onset of puberty [5]. Extended treatment was also associated with improved body proportions; one analysis found that after three years, treated children had a significantly lower upper-to-lower body segment ratio (indicating relatively longer limbs) compared to untreated achondroplasia controls [5].

Safety data from the extension trial continue to be favorable. With more than 460 patient-years of vosoritide exposure analyzed, there have been no drug-related serious adverse effects or deaths [5]. The observed adverse events were similar to those reported in the first year of treatment, including injection site reactions, brief blood pressure drops, and mild headaches. Taken together, the extension studies support the durability of vosoritide’s growth-promoting effect and suggest that longer-term therapy may offer additional benefits in skeletal proportionality while maintaining a tolerable safety profile [5].

Phase II Study in Children Younger than Five Years With Achondroplasia 

Children aged 3-59 months were treated with vosoritide to assess the impact of early initiation of therapy, given that growth deficits in achondroplasia occur starting in utero [27]. In this multinational, placebo-controlled trial, participants across 16 sites were divided into three cohorts by age (0-5 months, 6-23 months, and 24-59 months) and were given daily subcutaneous injections of vosoritide (30.0 µg/kg for infants aged 3-23 months; 15.0 µg/kg for children aged 24-59 months) [27]. While safety findings aligned with those of previous trials, the increase in height z-score was modest (0.25, 95% CI: -0.02 to +0.53), particularly in participants under the age of 24 months [27]. Although the absolute growth gain (~0.8-0.9 cm/year over placebo) was smaller than that observed in older children, it remained statistically significant and aligned with expectations, especially given the greater measurement variability in very young children [13]. Importantly, no adverse effects on developmental milestones were noted, and data supported the safety and potential benefit of initiating vosoritide treatment during infancy [27].

Further exploratory analyses indicated improvements in some craniofacial measures, including growth of the foramen magnum and midface structures [27]. Treated infants showed greater increases in facial bone and sinus volumes compared to placebo, which may translate into a reduced risk of middle ear infections and sleep apnea [27]. These findings, while preliminary, suggest that early vosoritide therapy might offer health benefits beyond increased stature and that further research into these effects is warranted.

Real-world clinical outcomes and post-trial experience

Early reports of clinical use of vosoritide have provided additional evidence of the drug’s effectiveness and safety in practice. Real-world studies in achondroplasia research are valuable for confirming that clinical trial outcomes in the general population and for capturing outcomes not fully assessed in trials, such as functional improvements or effects on comorbid conditions. Table 5 summarizes findings from post-clinical trial studies, including early access programs (EAPs), retrospective cohorts, case reports, and observational follow-up studies.

**Table 5:** Real-world and observational studies of vosoritide ACH = achondroplasia-specific growth reference; AGV = annualized growth velocity; 6MWT = 6-minute walk test; mos = months; y = years; AE = adverse event; SAE = serious adverse event; BMI = body mass index. FMS = foramen magnum stenosis.

Study	Setting and Design	Patients (Age Range)	Follow-up	Main Findings (Growth and Outcomes)	Safety Outcomes
Cormier-Daire et al., 2025 [16]	French early access program. Retrospective observational study.	57 patients (≥five years)	One year	AGV ~6.0 cm/yr; ~+6.2 cm height gain in one yr; height z-score +0.38 – growth effect mirrors Phase III trial. No new proportional changes noted at one year.	All AEs were mild (injection site reactions most common); no discontinuations; no treatment-related SAEs.
Reincke et al., 2025 [17]	Single center, Cologne, Germany. Retrospective observational study.	34 patients (2.8–15.3 years)	One year	Height z-score +0.52 (ACH charts), +0.38 (CDC charts) in one year (p	No serious drug-related AEs; profile like clinical trials (injection reactions, transient hypotension). No new safety concerns noted.
Sawamura et al., 2025 [18]	Single center, Japan. Prospective observational study.	17 patients (mean age 7.6 years)	One year	Height ↑ ~5.4 cm in one year. Spine and leg alignment improved: lumbar lordosis ↓ ~5°, mechanical axis of legs ↑ ~3.4° (less varus). Statistically significant shifts toward normal spinal alignment via reduction in exaggerated lordosis and genu varum.	No significant AEs reported. Study focused on radiographic outcomes; safety described as consistent with known profile.
Ireland et al., 2025 [22]	Single center, Australia. Case report.	One child (started therapy at four years)	One year	Independence in daily activities improved markedly: WeeFIM scores ↑ far above expected. Mobility domain +9 (vs +1 for average height peer); child achieved higher self-care independence vs age-matched achondroplasia peers. Suggests functional gain from height increase.	No adverse events reported. Child and family tolerated daily injections well.
Gunji et al., 2025 [23]	Single center, Nihon University School of Medicine, Japan. Case report.	One child (started therapy at two months of age)	14 months	Height ↑ ~15.5 cm in one year. AGV exceeded untreated ACH norms.	Early vosoritide therapy did not prevent worsening of FMS.
Clinton et al., 2025 [19]	Single center, Brazil. Case series.	18 children (two to ten years old)	5-17 months	No clinical outcomes related to growth were reported.	61.1% (11/18) developed hypertrichosis on the face, limbs, trunk, and back. Hair regrew upon reinitiation of treatment after temporary discontinuation in two patients.
Nishioka et al., 2024 [20]	Single center, Japan. Case series.	Two children (one month and three months)	Six months following observation of adverse reactions	No clinical outcomes related to growth were reported.	Both cases demonstrated that cardiovascular adverse events could occur in very young infants receiving vosoritide. Symptoms were reversible, and timing of feeding relative to injection was critical.

French Early Access Program (EAP)

France established nationwide EAPs for vosoritide in mid-2021, allowing children with achondroplasia to receive the drug before commercial availability. Cormier-Daire et al. reported results from this French EAP, which included 62 children aged 5-13 years enrolled across six centers within the French national rare disease reference center for constitutional bone diseases network [16]. Of these, 57 began vosoritide treatment (15 µg/kg daily), 38 completed six months of treatment, and 22 completed 12 months of treatment [16]. The growth outcomes in the French EAP were consistent with those from the Phase III trial: after one year of therapy, participants had a mean AGV of 6.0 cm/year, a statistically significant improvement from their pre-treatment velocity [16]. The average height gain was 6.2 cm over the 12 months [16]. Expected growth in children with achondroplasia at these ages is typically closer to 3-4 cm/year. Mean height z-scores in the EAP increased by +0.38 standard deviations against average-stature norms in one year [16]. No serious hypotensive episodes or allergic reactions were observed, and all reported adverse events were mild [16]. The EAP provided crucial evidence that a coordinated national program can successfully deliver daily vosoritide therapy to patients outside the controlled environment of a clinical trial.

German Retrospective Cohort

Reincke et al. published a 12-month retrospective observational study from a specialized skeletal dysplasia clinic in Germany. The study included 34 children and adolescents with achondroplasia (mean age: 8.7 years; range: 2.8-15.3 years) who were prescribed vosoritide [17]. Height measurements after one year of treatment reinforced vosoritide’s efficacy in promoting linear growth: height SDs increased significantly over 12 months, by an average of +0.52 (±0.35) on achondroplasia-specific growth charts and +0.38 (±0.44) on general population charts (both p < 0.0001) [17]. Most patients demonstrated an annual growth velocity above the untreated achondroplasia average for their age [17]. This study also examined body proportionality and other anthropometrics and found no significant changes in BMI, head circumference percentile, or upper-to-lower segment ratio after one year [17]. The lack of change in the upper/lower segment ratio at one year aligns with earlier clinical trial data, which showed that proportion improvements typically occur after several years of continuous treatment [5].

Unlike other trials to date, Reincke et al. performed assessments of physical function periodically throughout the study. The researchers took baseline measurements for the six-minute walk test (6MWT), a standardized indicator of functional capacity, and found significantly decreased baseline results compared to average height peers for patients with achondroplasia (mean z-score: −2.0 ± 1.1 SD vs average for unaffected peers) [17]. After 12 months of treatment with vosoritide, the researchers repeated the 6MWT testing showed a significant improvement from baseline, with the mean z-score improving from −2.0 SD to −1.39 SD [17]. As in previous studies, the authors noted a mild side effect profile with no serious adverse events attributed to vosoritide use [17]. The outcomes of this study reinforce that, in a real-world clinical setting, vosoritide can enhance growth and may help improve the development of physical function.

Prospective Study on Orthopedic Outcomes and Body Alignment

The short stature seen in achondroplasia is often accompanied by spinal and lower limb alignment issues, such as lumbar lordosis and genu varum, which can lead to chronic pain or the need for surgical correction in many patients [28]. A 12-month retrospective study performed in Japan, including 17 children with achondroplasia aged three to 12 years who were being treated with vosoritide, reported similar findings to previously described prospective studies and clinical trials, showing an increased average growth velocity of 5.4 ± 1.3 cm/year and minimal side effects [18].

The researchers obtained radiographs of the patients at baseline and after one year to measure spinal and lower limb alignment. The findings were encouraging: after one year of vosoritide treatment, a modest but statistically significant improvement toward normal alignment in several measurements was observed. The average lumbar lordosis angle decreased by ~5°, indicating correction of the swayback deformity commonly observed in patients with achondroplasia [18]. The mechanical axis angle of the femur, a measure of bow-leggedness, improved by an average of 3.4° toward neutral, and related femur/tibia angle metrics also shifted favorably [18]. Together, these changes demonstrate progress toward straightening of the lower limbs and normalization of spinal curvature. Improved alignment could translate to reduced back pain and less need for osteotomy to correct genu varum. Although extended follow-up studies are needed to assess the durability of these orthopedic outcomes, these findings have the potential for additional quality-of-life improvements beyond what height gain alone can provide [18].

Case Report: Functional Independence

Ireland et al. described a case of a five-year-old Australian boy with achondroplasia who was started on vosoritide and followed for functional progress over 12 months [23]. Using the WeeFIM (functional independence measure for children) instrument, researchers tracked his self-care, mobility, and cognitive independence relative to age-matched norms and historical data from peers with achondroplasia. After one year of therapy, the child not only grew in height but also showed accelerated gains in functional independence. Remarkably, his improvement in total WeeFIM score over the year was nearly three times greater than expected for an average-stature child between ages four and five, and far exceeded the typical gains seen in untreated children with achondroplasia [23]. Mobility domain scores, assessing competence in activities such as transfers and locomotion, increased ninefold more than what average unaffected peers are expected to achieve in that period [23]. The boy’s parents reported that he was able to perform tasks more independently and had better endurance [23]. This case provides preliminary evidence that enhanced growth, particularly in limb length, can positively impact a child’s ability to move and care for themselves.

Other Case Reports: Adverse Events

Three other case reports were identified describing unique side effects that were not observed during clinical trials. The first describes two infants with achondroplasia who experienced cardiovascular side effects within an hour after vosoritide injection. One of the infants developed hypotension, lethargy, and vomiting; the other exhibited pallor and tachycardia requiring IV fluids. In both cases, symptoms were linked to the timing of injections relative to feeding. Adjusting the interval between feeding and dosing (1.5-2 hours) reduced recurrence [20].

A separate case report documents the development of hypertrichosis in 11 out of 18 patients undergoing vosoritide therapy for achondroplasia. The children exhibited excessive hair growth in areas such as the back, arms, and legs after several months of treatment. This side effect had not previously been linked to vosoritide [19].

A third case report describes an infant with achondroplasia who developed worsening foramen magnum stenosis (FMS) during early vosoritide treatment. The patient, initially showing signs of FMS at two months of age, began vosoritide therapy at three months and demonstrated an increase in height gain. However, serial brain MRIs revealed progression from mild FMS without spinal cord abnormalities to spinal cord compression with abnormal medullary signal. This finding suggests that, contrary to earlier clinical trials, vosoritide therapy was not protective against the progression of FMS in this patient [22].

Safety profile of vosoritide

Vosoritide has consistently demonstrated a favorable safety and tolerability profile in clinical trials and real-world settings. This section summarizes key safety considerations.

Local Injection Site Reactions

Vosoritide is administered as a once-daily subcutaneous injection. Injection-site reactions are the most common side effect. In clinical trials, most patients experienced some form of local injection-site reaction, including erythema, itching, transient rashes, and swelling [5,7,27,29]. Symptoms are generally mild and self-limited and tend to lessen over time as patients and caregivers gain experience in site rotation and injection technique [30].

Systemic Allergic and Immunogenic Responses

Vosoritide is a recombinant 39-amino acid analog of C-type natriuretic peptide (CNP). Endogenous CNP is short-lived, with a half-life of 2.6 minutes, requiring significant modification of the original peptide to produce a compound stable enough for once-daily dosing [25]. Modified endogenous peptides have the potential to trigger host immune responses; however, no significant anti-drug antibodies impacting efficacy or patient safety have been detected in vosoritide-treated patients. Several patients developed low-titer, non-neutralizing antibodies with no observed clinical effect [31]. Our search yielded no reported cases of anaphylactic reactions. CNP signaling pathways, including those activated by vosoritide, can cause systemic vasodilation, leading to a transient drop in blood pressure accompanied by a baroreceptor-mediated increase in heart rate [20]. In clinical trials, about half of the patients experienced an asymptomatic decrease in blood pressure, typically within 60 minutes of injection [7,27,29]. A small subset of patients reported brief episodes of dizziness or lightheadedness. No episodes of sustained hypotension have been reported, nor have any chronic cardiovascular effects [7].

Bone Health and Disproportion

Given the stimulatory action of vosoritide on chondrocytes, concerns were raised within the achondroplasia patient community and advocacy groups during drug development about the potential for overgrowth in specific skeletal regions or worsening of disproportional features. Contrary to this, vosoritide’s action appears to predominantly normalize growth plate activity rather than cause excessive growth, with no reported evidence of abnormal skeletal changes [5,18]. Ongoing follow-up into adulthood will confirm final outcomes and monitor for any unanticipated effects on growth plate fusion or bone quality. Thus far, treated patients have demonstrated normal bone maturation [17].

Hypertrichosis

Clinton et al. followed 18 children with achondroplasia living in Brazil who were being treated with vosoritide [19]. The researchers observed the development of “pigmented, thin, and short hair on the face, arms, abdomen, back, and legs” in 11 of the patients receiving treatment [19]. Notably, this hypertrichosis resolved in all cases within one month of discontinuing the drug but returned in patients who resumed treatment [19]. No other studies in our review reported this side effect.

Mortality

No deaths related to vosoritide have been reported. One mortality was reported in the Phase II under-five-year-old trial, which was deemed a case of sudden infant death syndrome (SIDS) unrelated to vosoritide treatment [27]. No signals of increased mortality or life-threatening adverse outcomes have been observed in any age group. 

Limitations of current data and future directions

Despite promising results, several limitations exist regarding the current data on vosoritide. Long-term outcomes into adulthood remain unknown. Vosoritide use has been studied in controlled trials lasting up to two years, in retrospective and prospective studies up to one year, and open-label extensions up to seven years, but no data have followed treated patients to their final adult height. It is therefore uncertain how much height gain vosoritide can ultimately provide, or whether early improvements in growth velocity translate into a significant difference in mature adult stature [7]. It is also unclear whether long-term therapy might result in delayed adverse effects on growth plate biology. No such effects have been observed, and studies show that bone age progression appears normal with vosoritide treatment [13,18].

The current studies also include relatively small sample sizes, limiting statistical power, especially for subgroup analyses. This limitation was evident in the infant trial, where only trends were observed with relatively wide confidence intervals and a lack of statistical significance. As noted by investigators, a 1 cm/year gain in an infant may be difficult to detect due to measurement variability [27]. Therefore, the modest growth benefit seen in the under-five trial may be an underrepresentation of what more extensive studies could demonstrate.

Another consideration is the focus on linear growth metrics as primary outcomes, which may not capture the full clinical impact of therapy. Height, velocity, and height z-score are convenient quantitative endpoints, but improvements in proportions, functionality, and complication avoidance are also important to consider. Several studies attempted to capture these less-reported outcomes but did so using diverse measurement tools, making cross-study comparisons difficult. Future research should aim to quantify additional variables that are relevant to the functionality and quality of life of children with achondroplasia.

## Conclusions

Vosoritide has the potential to be a landmark therapy for achondroplasia, demonstrating enhanced osteochondral growth in children with a genetic condition that was previously considered untreatable. In multiple pediatric clinical trials, vosoritide has consistently increased growth velocity and height in children with achondroplasia, with treated individuals growing an average of 1.5-2.0 cm/year faster than untreated peers. These gains, sustained over several years of follow-up, demonstrate that vosoritide can narrow the growth deficit caused by achondroplasia. Preliminary clinical observations have thus far confirmed the growth-promoting effects of vosoritide and have also shown benefits in other measures such as functionality and independence, spinal and lower limb alignment, and endurance. The drug has a favorable safety profile, with only transient to mild side effects and no reported serious long-term safety concerns. This balance of efficacy and safety led to vosoritide becoming the first evidence-based medication available to increase height in children with achondroplasia to gain regulatory approval in numerous countries worldwide. Future research should aim to inform best practices for vosoritide’s use and determine its role in the early treatment of achondroplasia.
